# A Murine Bispecific Antibody Efficiently Redirects T Cells Against *Calr* Mutated Stem Cells In Vivo

**DOI:** 10.1002/ajh.70206

**Published:** 2026-01-14

**Authors:** Shengen Xiong, Tamara Wais, Cecilia Varga, Christina Schueller, Sarada Achyutuni, Robert Kralovics

**Affiliations:** ^1^ Department of Laboratory Medicine Medical University of Vienna Vienna Austria

## Abstract

Calreticulin (CALR) mutations are prevalent in 20%–30% of patients with BCR::ABL1‐negative myeloproliferative neoplasms (MPN). Mutant calreticulin (mutCALR), presented by the thrombopoietin receptor (MPL, also known as TPOR or CD110) on the surface of the disease‐initiating MPN progenitors, represents an ideal target for curative immunotherapies including monoclonal antibodies, bispecific T cell engaging antibodies (TCE), and CAR‐T cell therapies. Despite that two clinical TCE candidates have advanced into phase 1 trials in recent 2 years, depletion of mutCALR+ hematopoietic stem cells and normalization of hematopoiesis remained absent in preclinical evaluation. Here, we developed a bispecific T cell engager DX1‐2C11 that specifically and efficiently eradicates mutCALR‐expressing cells via recruiting polyclonal T cells. DX1‐2C11 depleted Ba/F3 cells expressing mutCALR, as well as primary murine myeloid cells in a dose‐dependent manner in vitro. In CALRdel52 transgenic mice, a single dose of DX1‐2C11 activated CD4^+^ and CD8^+^ T cells in the peripheral blood, spleen and bone marrow within 24 h. Furthermore, a single dose of DX1‐2C11 reduced platelet counts in the periphery and decreased mutant stem/progenitor cell populations in the spleen and bone marrow by Day 7 posttreatment. Notably, the reduction of mutant burden was durably maintained in secondary recipient mice. In the disseminated NSG model, DX1‐2C11 delivered immediate tumor burden reduction and significantly prolonged the overall survival of mice compared to the control group. Taken together, these data suggest that bispecific T cell engaging antibody targeting mutCALR represents a curative strategy that efficiently eliminates mutant MPN stem cells in vivo.

## 1 Introduction

Philadelphia‐negative or BCR::ABL1‐negative myeloproliferative neoplasms (MPNs) are a spectrum of chronic hematologic malignancies that originate from somatic mutations in hematopoietic stem and progenitor cells (HSPCs). Over 90% of the MPN patients are associated with driver mutations of the JAK/STAT pathway, primarily in the *JAK2* [1, 2, 3, 4, 5], *CALR* [6, 7], and *MPL* [8, 9, 10] genes. These mutations lead to clonal expansion of the mutant progenitors and overproduction of mature blood cells of the myeloid lineage, manifesting as excessive red blood cells (polycythemia vera, PV), platelets (essential thrombocythemia, ET), and/or fibrosis‐producing white blood cells (primary myelofibrosis, PMF) [11]. Whereas PV and ET are relatively indolent phenotypes, they may progress to myelofibrosis as a complication. Further transformation to secondary acute myeloid leukemia represents a major cause of mortality among MPN patients [12]. Conventional treatment options for MPN patients deliver symptom control to a certain extent but fail to eradicate MPN stem cells or prevent disease progression.

Calreticulin (CALR) mutations are the second most frequent (20%–30%) in all MPNs [6, 7, 13, 14]. These are +1/−2 frameshift mutations in the *CALR* exon 9, resulting in the loss of the KDEL endoplasmic reticulum retention signal and the generation of an identical mutant C‐terminal neoantigen which allows its aberrant interaction with the thrombopoietin receptor (MPL) [15, 16, 17, 18, 19, 20, 21]. To date, over 150 distinct *CALR* indel variants have been reported, but most are rare [22, 23, 24, 25]. The type 1 mutation, CALRdel52 (a 52 bp deletion), and type 2 mutation, CALRins5 (5 bp insertion) are the most common types present respectively in ~50% and ~30% of all CALR mutated cases [22].

Since the mutant calreticulin (mutCALR) driven cancer signaling relies on its membrane presentation by MPL on the surface of the MPN progenitors [15, 16, 17, 18, 19, 20, 26], it constitutes a rational and tumor specific immunotherapy target for *CALR* mutated patients [27, 28]. Previous studies have largely pursued a therapeutic strategy of targeting CALR mutants with anti‐mutCALR monoclonal antibodies (mAbs), designed either to antagonize the CALR/MPL‐mediated oncogenic signaling [29, 30, 31] or to eliminate the mutCALR positive HSPCs through antibody induced cytotoxic mechanisms [32, 33]. However, due to the low expression level of MPL, T cell redirection strategies including T cell engaging antibodies (TCE) and CAR‐T cell therapies might be more sensitive than antibody effector functions in eliminating the MPN progenitors [34, 35]. In recent years, bispecific T cell engaging antibodies represent an ideal drug modality outperforming CAR‐T cells and challenging frontline antibody treatments in many hematological malignancies [36, 37, 38]. Three mutCALR specific drug candidates—an antagonistic mAb (INCA033989) [30, 31] and two TCEs (JNJ‐88549968 and INCA035784) [39, 40]—have advanced into phase I clinical trials in the last 2 years. However, a direct proof‐of‐concept study demonstrating the depletion of CALR mutant HSPCs is missing in the preclinical datasets for the two TCE candidates, as both primarily relied on synthetic xenograft models in immunodeficient mice, thus necessitating a preclinical TCE platform to elucidate how CALR mutated HSPCs are recognized and eliminated by autologous mouse T cells in an immunocompetent setting.

Here, we report the development of a murine TCE platform and a bispecific T cell redirecting antibody DX1‐2C11 that selectively targets mutCALR positive cells in vitro and in vivo. DX1‐2C11 activated autologous mouse T cells and eliminated the mutCALR expressing MPN stem cells efficiently in transgenic mouse models. Our data demonstrate the feasibility of developing T cell engagers for the treatment of mutCALR positive MPN with the potential to be a curative therapy.

## 2 Methods

A complete description of methods is presented in supplemental methods (Appendix S1).

### 2.1 Design and Production of Bispecific T Cell Engagers

The bispecific murine TCE platform in the Fab‐Fc/ScFv‐Fc mouse IgG2a format was designed based on insights from structural homology. The TCEs were engineered with the mouse equivalent “knob‐into‐hole” and Fc silencing mutations to ensure efficient heavy chain pairing and to prevent killing from the Fc medicated effector functions (see Table S1). Detailed methods for TCE vector cloning, protein production and purification are described in supplemental methods (Appendix S1).

### 2.2 T Cell Redirection Assay

Mouse T cells were isolated from mouse splenocytes using a commercial kit (MojoSort Mouse CD3 T cell Isolation kit, 480099), and expanded for 3–5 days following the manufacturer's instructions (Dynabeads Mouse T‐Activator CD3/CD28 for T‐Cell Expansion and Activation kit, Gibco). The beads were removed and T cells were rested in culture for an additional 2–3 days. Ba/F3 cells were cocultured with T cells for 72 h at an effector‐to‐target ratio of 3:1 with DX1‐2C11 or isotype control molecules. T cell responses were measured as the proportion of live CD8^+^ or CD4^+^ T cells expressing CD25, CD69, CD107a, and IFNγ. Killing of CALR mutants was measured as the decrease in the remaining live target cell count after treatment normalized with the untreated and noneffector cell conditions.

### 2.3 Ex Vivo Killing Assay

The killing assay was performed on bone marrow cells (BMCs) isolated from C57BL/6N mice treated with DX1‐2C11 or isotype control for 2 days in StemSpan SFEM medium with supplements. BMCs were seed at a density of 1 × 10^5^ to 2 × 10^5^ BMCs per 1 mL medium per well in the 6‐well plates. DX1‐2C11 or TCE control was added at 1, 10, 100 nM to the wells in triplicates. Flow cytometry analysis were used to quantify T cell activation, degranulation and cytokine release measured as the proportion of live CD8^+^ T cells expressing CD25, CD69, and CD107a. Tumor cell killing was calculated as the percentage of 7AAD positive CD45.2^+^ BMCs.

### 2.4 Treatment of VavCre CALRdel52/+ Mice

The efficacy of DX‐2C11 was evaluated in an immunocompetent C57BL6/N mouse model as previously described [32]. On Day −14, 10 female VavCre CALR^del52/+^ mice (18–20 weeks old) were randomized into two groups by body weight and platelet counts. The mice were intraperitoneally injected with a single 10 mg/kg dose of TCE or isotype control on Day 0 and then euthanized on Day 7 for blood counting and organ harvesting. Single cell suspension of splenocytes and bone marrow cells was made from each mouse for quantification of the absolute number of LSKs, ST‐HSCs, and LT‐HSCs via flow cytometry.

### 2.5 Treatment of Competitive Bone Marrow Transplanted Mice

Bone marrow cells from 12‐ to 24‐week‐old donor C57BL6/N mice from the VavCre CALR^del52/+^ CD45.2 background and the wild type CD45.1 background were mixed in a 1:1 ratio before transplantation. CD45.1 wild type recipient mice received two times 5.5Gy irradiation, 16 h apart. Peripheral blood was drawn biweekly starting from 30 days after bone marrow transplantation for blood counting and flow cytometric measurement of CD45.1/CD45.2 chimerism of T cells (CD3ε^+^), B cells (CD19^+^), granulocytes (Gr1^+^), and myeloid cells (CD11b^+^). Single cell suspension from spleen and bone marrow tissues was prepared for the measurement of CD45.1/CD45.2 chimerism within the LSK, ST‐HSC, and LT‐HSC compartments. T cell activation was measured 48 h post‐TCE treatment in an independent cohort of mice of the same experimental settings.

### 2.6 Treatment of Immunodeficient NSG Mise With Disseminated CALR Mutant Cells

The in vivo study for long‐term efficacy evaluation was performed in 8–12‐week‐old immunodeficient NSG (NOD.Cg‐Prkdcscid Il2rgtm1Wjl/SzJ) mice (5 mice per group). On Day 0, 0.5 million Ba/F3‐MPL/CALRdel52 cells expressing luciferases were inoculated intravenously. Each mouse received an intraperitoneal injection of 10 mg/kg human IVIG on Day 3 for Fc receptor saturation followed by an intravenous injection of 10 million mouse T cells on Day 4. The mice were treated with 10 mg/kg of DX1‐2C11 or isotype control on Days 5, 7, 9, 12, 15, 18, and 21. Bioluminescence imaging (using LagoX), body weight tracking, and survival were monitored throughout the course of the experiment.

### 2.7 Statistical Analysis

Statistical analysis and graphs were generated using GraphPad Prism software (GraphPad Software, La Jolla, CA) and R (R Foundation, Vienna, Austria). A detailed description can be found in Supporting Information.

## 3 Results

### 3.1 Design and Biophysical Characterization of DX1‐2C11

**FIGURE 1:**
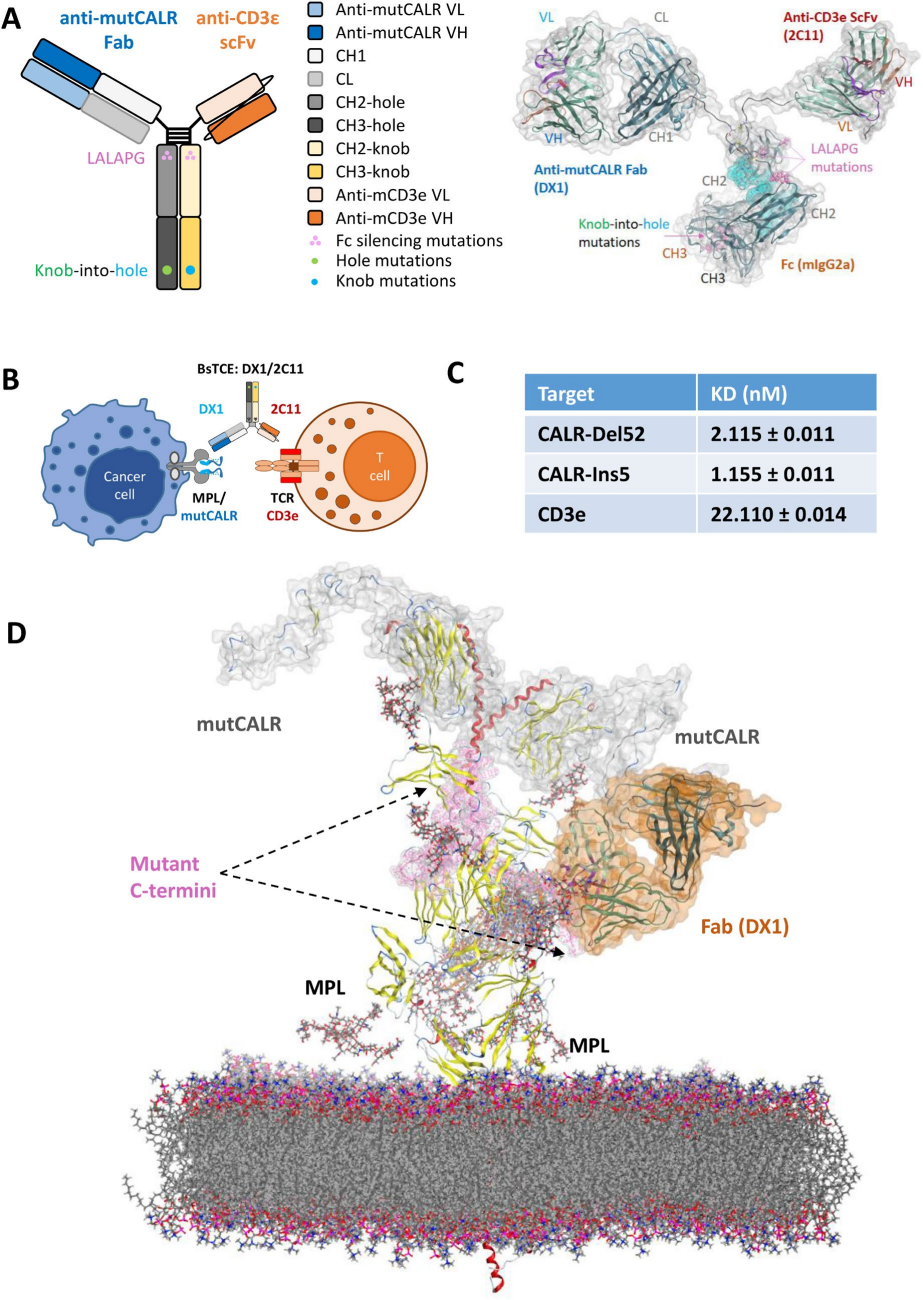
Structural design and biochemical properties of bispecific T cell engager DX1‐2C11. (A) Schematic design and 3D modeling of bispecific T cell engager DX1‐2C11 with a Fab (DX1) targeting mutCALR, a scFv (145‐2C11) targeting mouse CD3ε, and a mouse IgG2a Fc engineered with effector function silencing (L234A, L235A, P329G) and heterodimeric pairing mutations (knob‐into‐hole). (B) Schematic mode of action of DX1‐2C11 by inducing synthetic immune synapses between T cells and mutCALR+ MPN cells. (C) The binding affinity of DX1‐2C11 to CALR‐del52, CALR‐ins5 and mouse CD3ε measured by biolayer interferometry using Octet Red96e. KD, the equilibrium dissociation constant. (D) Epitope accessibility of the CALR mutant tail simulated by in silico modeling and docking using AlphaFold 2 and RosettaDock. DX1 Fab (in orange) docked to the C‐terminus (in pink) of mutCALR (in white) exposed on the MPL/TPOR tetrameric complex. [Color figure can be viewed at wileyonlinelibrary.com]

To enable preclinical evaluation of TCE drug candidates in immunocompetent mouse models, a universal murine TCE platform is necessary. Based on insights from structural analysis, we designed the TCEs in the asymmetric Fab‐Fc/ScFv‐Fc format with an anti‐mutCALR Fab arm (clone DX1), a monovalent anti‐CD3ε ScFv arm (clone 145‐2C11 [41, 42]) (Figure 1A). Furthermore, we generated an Fc domain that contained the mouse equivalent of “knob‐into‐hole” mutations [43] to ensure correct heavy chain pairing as well as the “LALAPG” mutations [44] for Fc silencing (Figure 1A, Table S1). The mode of action of TCEs is to induce the formation of a synthetic immune synapse between polyclonal T cells and target cells, as depicted in Figure 1B. TCEs were produced through transient transfection of Expi293F cells and then purified using protein A chromatography followed by ion exchange and size exclusion chromatography polishing. Native‐PAGE gel electrophoresis confirmed the high purity of DX1‐2C11 after purification (Figure S1A).

Next, we determined the binding affinity of DX1‐2C11 to recombinant CALR‐del52, CALR‐ins5, and mouse CD3ε by biolayer interferometry. DX1‐2C11 displayed nM level affinities to CALR‐del52 (KD = 2.115 nM), CALR‐ins5 (KD = 1.155 nM), and mouse CD3ε (KD = 22.110 nM) (Figure 1C, Figure S1B, Table S2).

### 3.2 DX1‐2C11 Selectively Binds to the c‐Terminus of mutCALR

To verify the epitope of DX1 and its structural accessibility in the MPL/mutCALR tetramer on cell membrane, we performed in silico docking of the DX1 Fab arm to mutCALR and also the MPL/mutCALR tetrameric complex structure previously reported [20]. As predicted by the docking sites to the MPL/mutCALR complex with minimum energy, the epitope of DX1 is a linear motif in the C‐terminus of mutCALR (Figure 1D). All top poses docked to the mutCALR monomer were clustered to the same C‐terminal docking site (Figure S1C), which further consolidated the prediction. Alanine‐scanning ELISA using a peptide library confirmed the “CLQGW” motif (Figure S1D) as the predicted epitope shared by all mutant CALRs.

### 3.3 DX1‐2C11 Induces Potent and Specific Killing of Ba/F3‐MPL Cells Expressing mutCALR In Vitro

**FIGURE 2:**
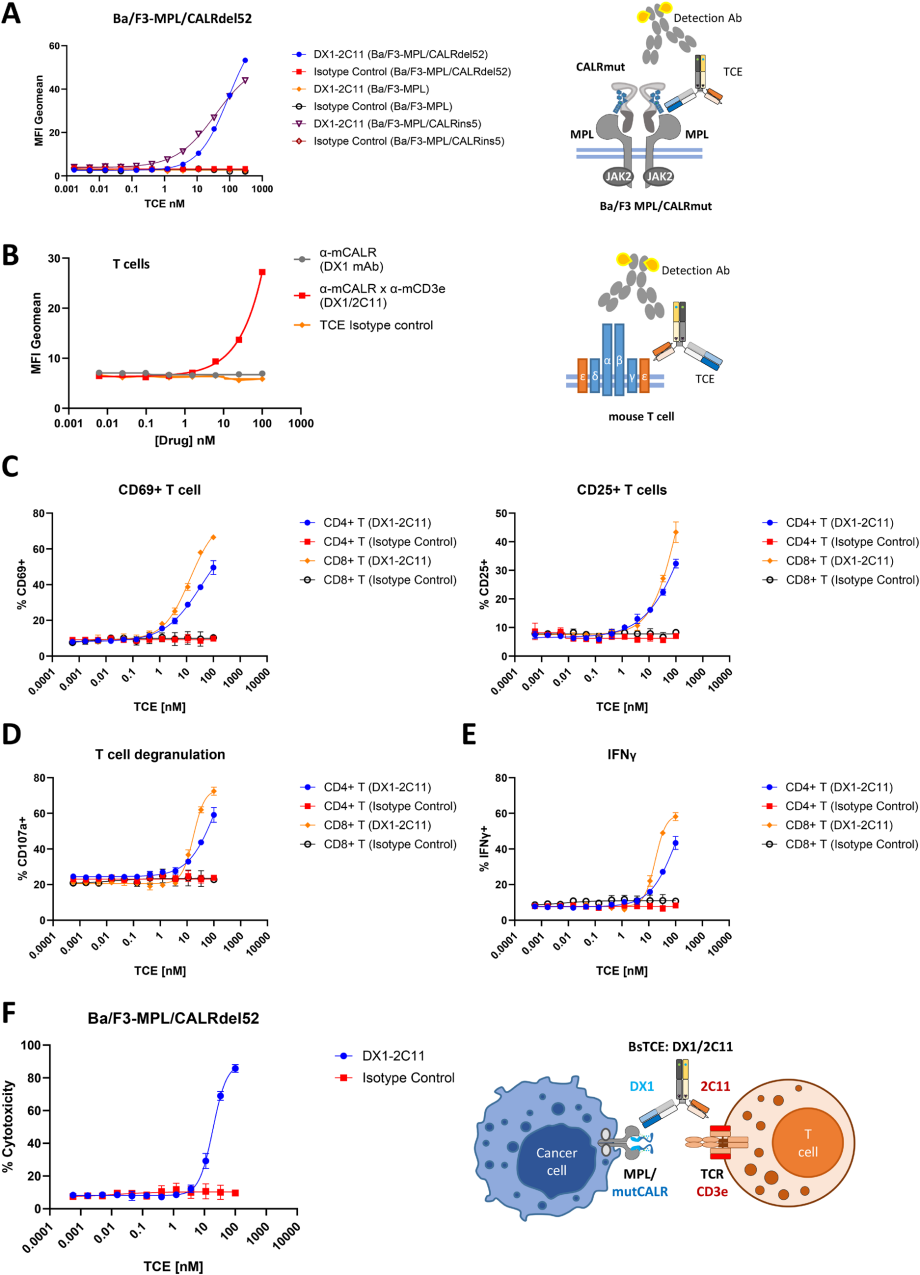
DX1/2C11 (α‐CALRmut × α‐mCD3ε) binds specifically to Ba/F3 cells expressing mutCALR and induces potent T cell mediated killing. (A) DX1‐2C11 specifically binds to mutCALR+ Ba/F3 cells. (B) DX1‐2C11 stained mouse T cells efficiently. (C–G) DX1‐2C11 mediated T cell cytotoxicity to Ba/F3‐MPL/CALRdel52 cells, (C) T cell activation, (D) T cell degranulation, (E) IFNγ production, and (F) Ba/F3 killing after treatment with DX1‐2C11 or control TCE molecules. Data represent the mean ± SD. [Color figure can be viewed at wileyonlinelibrary.com]

To confirm the binding specificity of DX‐2C11 to mutant calreticulins, we initially used Ba/F3 cells expressing MPL/CALRdel52 or MPL/CALRins5 complexes. The parental cell line (Ba/F3‐MPL) was included as a control. DX1‐2C11 specifically bound to CALRdel52 and CALRins5 on the surface of Ba/F3‐MPL/CALRdel52 or Ba/F3‐MPL/CALRins5 cells while maintaining absent on the surface of Ba/F3‐MPL cells at the highest concentrations (Figure 2A). Similarly, DX1‐2C11 specifically binds to mouse T cells while the TCE isotype control and DX1 monoclonal antibody do not (Figure 2B).

To test the T cell redirection and mutCALR+ cell killing, mouse T cells and Ba/F3‐MPL/CALRdel52 cells were cocultured in an effector‐to‐target (E:T) ratio of 3:1 in the T cell redirection assay. DX1‐2C11 treatment for 72 h has led to robust T cell activation, degranulation, cytokine release (Figure 2C–E) and Ba/F3‐MPL/CALRdel52 cell depletion in a dose‐dependent manner in vitro (Figure 2F). Similarly, comparable drug responses were observed to Ba/F3‐MPL/CALRins5 cells (Figure S2A–D), indicating that our TCE platform is not restricted to type 1 mutant and can accommodate diverse CALR mutant contexts.

To evaluate whether binding of DX1 mAb or DX1‐2C11 to mutCALR on the cell surface exerts an antagonistic effect on cell growth, both Ba/F3‐MPL and Ba/F3‐MPL/mutCALR cells were incubated with either DX1 or DX1‐2C11 for 72 h. Cell proliferation was subsequently assessed using the CellTiter‐Glo assay. No significant changes in cell growth were observed, indicating that neither DX1 nor DX1‐2C11 inhibited the growth of mutCALR+ Ba/F3 cells or mutCALR‐ Ba/F3 cells (Figure S3A,B). These findings demonstrate that the activity of DX1‐2C11 is exclusively mediated through T‐cell redirection.

Taken together, these data indicate that DX1‐2C11 is a potent bispecific anti‐mutCALR T cell engager that binds specifically to mutCALR and mouse CD3ε, mediating potent T cell‐dependent cytotoxicity against mutCALR expressing cells.

### 3.4 The Role of Soluble mutCALR in Anti‐mutCALR TCE Cytotoxicity

Mutant CALR proteins have been reported to be secreted by the CALR mutated progenitor cells [15, 32, 45, 46, 47]. Secreted mutCALR circulating in the plasma of the patients has been found in complex with soluble transferrin receptor 1 (sTR1) which functions as a carrier protein and increases the half‐life [15]. Level of secreted mutCALR in patient plasma has been reported to be up to 160 ng/mL and on average 25.64 ng/mL [15]. Thus, secreted mutCALR may function as a drug sink to diminish the efficacy of anti‐mutCALR immunotherapies, be it monoclonal antibodies, bispecific T cell engagers, or CAR‐T cells. To address this, we performed a TCE binding competition assay in the presence of increasing concentrations of recombinant CALRdel52. The competition was evaluated at 60 nM (semi‐saturating) and 300 nM (saturating) concentrations of DX1‐2C11. At higher CALRdel52 concentrations (> 20 nM [1032 ng/mL]), a reduction in DX1‐2C11 binding to Ba/F3‐MPL/mutCALR cells was observed, indicating concentration‐dependent inhibition of binding (Figure S3C).

To further evaluate the impact of secreted mutCALR on DX1‐2C11 mediated cytotoxicity, we assessed killing of Ba/F3‐MPL/mutCALR cells in the presence of increasing concentrations of recombinant CALR‐del52 (0, 10, 100, and 200 nM). Low (10 nM) and intermediate (100 nM) concentrations only modestly impaired killing, as reflected by an increase in IC_50_ from 11.78 to 18.17 nM and 351 nM, respectively, whereas 200 nM CALRdel52 substantially abrogated TCE activity (Figure S3D). Since the physiological levels of secreted mutCALR reported so far are about 0.5–3.2 nM, it is unlikely that secreted mutCALR in the patient plasma would significantly attenuate the killing efficacy of TCEs.

### 3.5 Physiological Expression of CALR‐del52 in Primary Murine Progenitor Cells Triggers T Cells Activation in the Presence of DX1‐2C11 In Vitro

**FIGURE 3:**
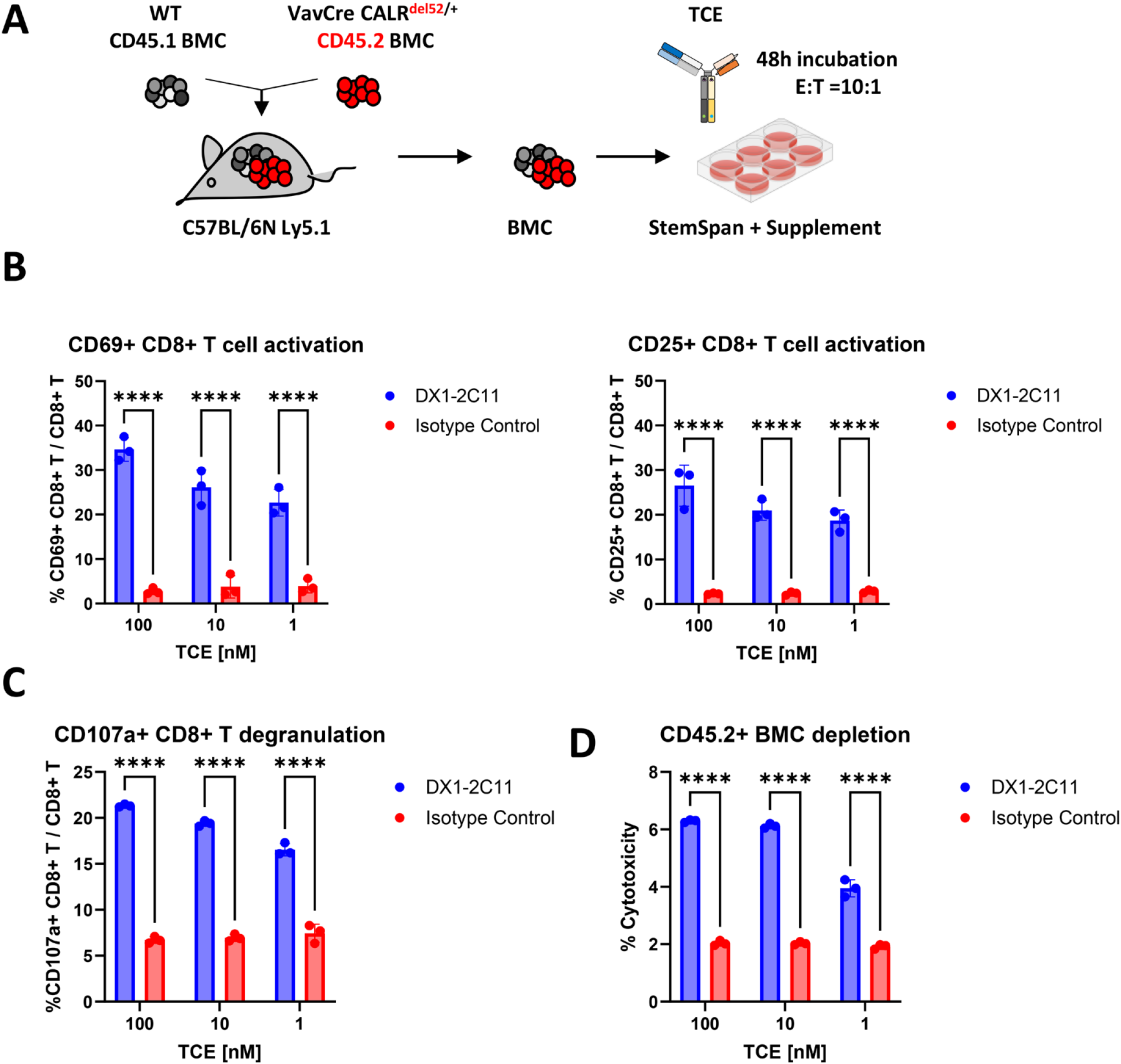
Anti‐mutCALR bispecific T cell engager DX1‐2C11 activates T cells and induces potent killing of mutCALR+ progenitor cells ex vivo. (A) Schematic experiment design. The chimerism of the VavCre CALR^del52/+^ CD45.2^+^ bone marrow cells reached 90% before drug incubation. (B) T cell activation at 24 h postincubation. (C) T cell degranulation at 24 h. (D) Cell lysis of CD45.2^+^ bone marrow cells. Significance was determined using a 2‐way ANOVA analysis. *p < 0.05, **p < 0.01, ***p < 0.001, ****p < 0.0001. Data represent the mean ± SD. [Color figure can be viewed at wileyonlinelibrary.com]

As DX1‐2C11 demonstrated potent and specific killing of Ba/F3‐MPL/mutCALR cells overexpressing both MPL and mutCALR, we next investigated if endogenous expression of MPL and CALRdel52 is sufficient to elicit TCE‐mediated T cell activation. To this end, we employed an immunocompetent transgenic mouse model that recapitulates an essential thrombocythemia (ET) phenotype characterized by progressive thrombocytosis and megakaryocytosis. This model harbors the chimeric mouse‐human CALR^del52^ allele driven by the *VavCre* promoter, enabling hematopoietic cell specific expression of mutCALR [32]. Bone marrow cells were isolated from recipient mice transplanted with VavCre CALR^del52/+^ CD45.2^+^ and wt CD45.1^+^ donor cells once the CD45.2/CD45.1 chimerism reached 90%. The isolated BMCs were treated with DX1‐2C11 or isotype control for 48 h (Figure 3A). As anticipated, T cell activation and degranulation were observed exclusively in DX1‐2C11‐treated cocultures (Figure 3B,C), which was accompanied by increased cell lysis in CD45.2^+^ bone marrow cells (Figure 3D).

### 3.6 DX1‐2C11 Delivered Potent Killing to mutCALR Positive LSKs, ST‐HSCs, LT‐HSCs In Vivo in VavCre CALR^del52^ Transgenic Mice

To investigate the in vivo efficacy of DX1‐2C11, we initially employed a competitive bone marrow transplantation model (Figure S4A). Bone marrow cells from *VavCre CALR* ^del52/+^ CD45.2^+^ and wild type CD45.1^+^ mice were mixed at a 1:1 ratio and transplanted into lethally irradiated CD45.1^+^ C57BL/6N recipients. After 30 days of bone marrow reconstitution, the mice were randomized by peripheral CD45.2/CD45.1 chimerism. These mice were treated with 10 mg/kg DX1‐2C11 or isotype control twice weekly for 31 days and were then sacrificed for the chimerism analysis in the hematopoietic compartments in spleen and bone marrow. To our dismay, no significant normalization of chimerism was observed (data not shown), which prompted us to hypothesize that the 2C11 module derived from Armenian hamster may be immunogenic even in the scFv format in immunocompetent mice, leading to the neutralization of DX1‐2C11 over time (Figure S4B). To verify this, we measured the titer of anti‐2C11 antibodies in the sera of treated mice. Strikingly, high titers of anti‐2C11 antibodies were detected in DX1‐2C11‐treated animals (Figure S4C), confirming that the 2C11 scFv arm is still highly immunogenic in vivo.

**FIGURE 4:**
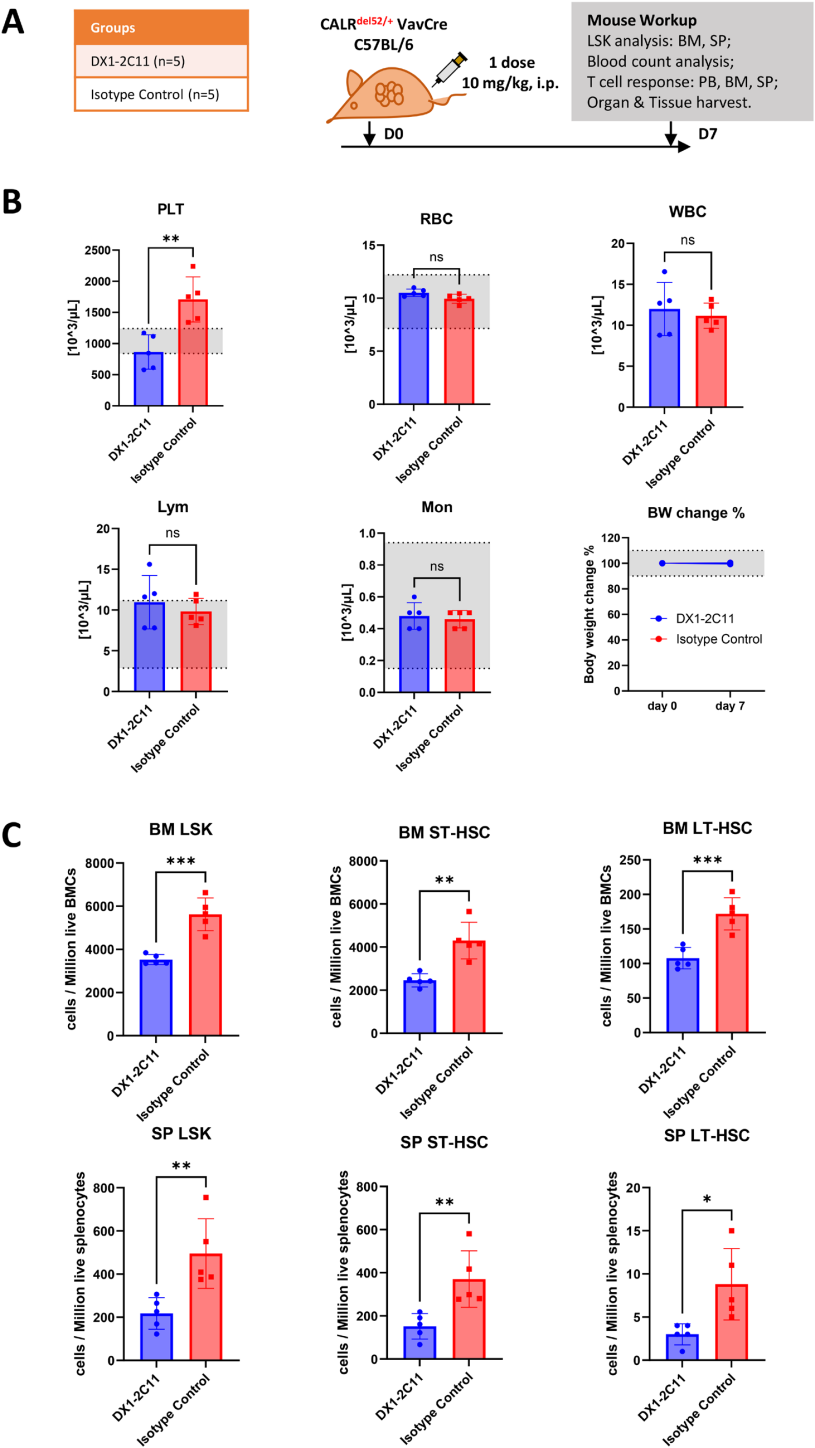
A single dose of DX1‐2C11 TCE induces the killing of mutant LSKs, ST‐HSCs and LT‐HSCs in VavCre CALR^del52/+^ mice. (A) Schematic design of the study to quantify mutant HSC depletion at 7 days posttreatment in VavCre CALRdel52/+ mice. (B) Blood count analysis. Gray area indicates normal range of blood counts. A single dose of DX1‐2C11 maintained to normalize the number of platelets (PLT) on Day 7 while red blood cells (RBC), white blood cells (WBC), lymphocytes (Lym), and monocytes (Mon) remain stable. Treatment of DX1‐2C11 does not influence body weight (BW). (C) DX1‐2C11 maintained to decrease the absolute number of LSKs, ST‐HSCs, LT‐HSCs in vivo on Day 7. Significance was determined using an unpaired *t*‐test. *p < 0.05, **p < 0.01, ***p < 0.001. ns, not significant. Data represent the mean ± SD. [Color figure can be viewed at wileyonlinelibrary.com]

Although long‐term evaluation of DX1‐2C11 in immunocompetent mice is not feasible due to its immunogenicity, short‐term efficacy evaluation remained informative. To assess whether DX1‐2C11 can induce T cell activation and deplete mutCALR expressing progenitors, VavCre CALR^del52/+^ mice were randomized to receive a single dose of DX1‐2C11 (10 mg/kg) or isotype control (Figure 4A). In one experiment, mice were euthanized 24 h after treatment to capture acute T cell activation (Figure S4D). Within this period, DX1‐2C11 activated 4.66%, 32.7%, and 32.3% of CD4^+^ T cells, and 8.45%, 18.8%, and 24.4% CD8^+^ T cells in peripheral blood, spleen, and bone marrow, respectively (Figure S4E). These data highlighted a stronger T cell response in the spleen and bone marrow compared to peripheral blood.

In a separate cohort, mice were sacrificed on Day 7 posttreatment to evaluate DX1‐2C11‐induced cytotoxicity toward hematopoietic cells (Figure 4A). A sustained reduction in platelet counts was observed in the DX1‐2C11‐treated group, whereas counts of red blood cells, white blood cells, lymphocytes, and monocytes remained unaffected (Figure 4B). To further assess the impact of DX1‐2C11 on HSPCs, we quantified LSKs (Lineage^−^Sca1^+^c‐Kit^+^), short‐term HSCs (ST‐HSCs), and long‐term HSCs (LT‐HSCs) in both spleen and bone marrow. All three populations were significantly reduced following DX1‐2C11 treatment (Figure 4C), indicating targeted depletion of mut*CALR*‐expressing progenitors in these tissues.

### 3.7 DX1‐2C11 Induced Specific and Durable Depletion of Mutant HSCs in Competitive Bone Marrow Transplantation Model

**FIGURE 5:**
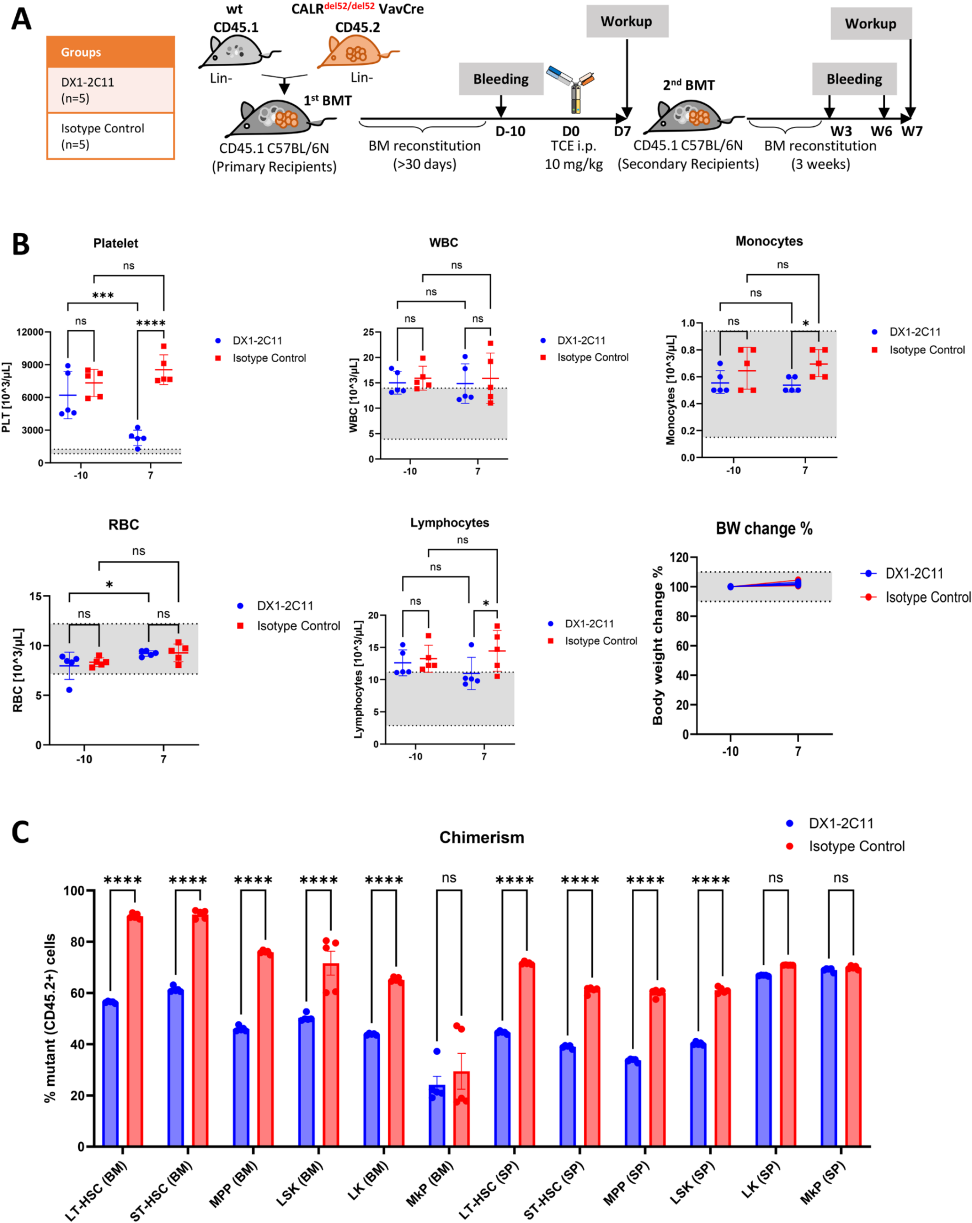
DX1‐2C11 selectively targets mutCALR^+^ (CD45.2^+^) HSPCs and MkPs in the competive bone marrow transplanted mice. (A) Schematic experimental design to compare CD45.2/CD45.1 chimerism in C57BL6/N recipient mice. The competitive bone marrow cell mixture from the VavCre CALR^del52/del52^ CD45.2^+^ and wt CD45.1^+^ bone marrow cells (1:1) were transplanted. The blood counts and chimerism were assessed biweekly after 30 days for mice randomization. Five million BMCs from each treated mice were transplanted to secondary recipient mice (CD45.1^+^ C57BL6/N, *n* = 10) for chimerism analysis. (B) DX1‐2C11 normalized platelet counts while the WBCs, RBCs, and lymphocytes remained unaffected. Gray area indicates normal range of blood counts. Data shown mean ± SD (*n* = 5 mice/group). (C) CD45.2/CD45.1 (mut/wt) chimerism analysis of hematopoietic stem and progenitor cells in bone marrow (BM) and spleen (SP). Significance was determined using a 2‐way ANOVA analysis. *p < 0.05, **p < 0.01, ***p < 0.001, ****p < 0.0001. [Color figure can be viewed at wileyonlinelibrary.com]

To verify selective elimination of mutant HSCs, TCE activity was further evaluated in a competitive bone marrow transplantation model. CD45.2^+^ VavCre CALR^del52/+^ and CD45.1^+^ wild type bone marrow cells were mixed at a 1:1 ratio and transplanted into CD45.1^+^ recipient mice. The mice were randomized once peripheral blood CD45.2/CD45.1 chimerism exceeded 80% (Day −10). A single dose of TCE (10 mg/kg) was administered on Day 0 and the mice were terminated on Day 7 (Figure 5A). DX1‐2C11 normalized platelet counts without significantly affecting RBC, WBC, or monocyte levels (Figure 5B). Notably, DX1‐2C11 induced a marked reduction of CD45.2^+^ cells in all progenitor compartments including LT‐HSC, ST‐HSC, MPP, and LSKs, resulting in significantly decreased CD45.2/CD45.1 chimerism, thereby confirming selective depletion of *CALR* mutant MPN progenitors (Figure 5C, Figure S5A,B).

To further assess selectivity and durability, 5 × 10^6^ bone marrow cells from each primary recipient mice were transplanted into lethally irradiated (11Gy) CD45.1^+^ C57BL6/N mice (12–24 weeks; *n* = 10) (Figure 5A). Sustained reductions in peripheral platelet counts (Figure S5C) and peripheral blood chimerism (Figure S5D) were observed at Weeks 3 and 6, and further divergence across HSPC compartments in the bone marrow and spleen was confirmed at Week 7 (Figure S5E). These findings substantiated highly specific and durable clearance of CALR‐mutant HSCs following a single administration of DX1‐2C11.

In summary, DX1‐2C11 exhibits robust short‐term activity in transgenic VavCre CALRdel52 models by promoting T cell activation and selectively depleting mutant hematopoietic progenitors. However, its long‐term treatment in immunocompetent mice is limited by anti‐drug antibody (ADA) responses directed against the immunogenic 2C11 component.

### 3.8 Evaluation of the Long‐Term Efficacy of DX1‐2C11 in a Disseminated NSG Mouse Model

**FIGURE 6:**
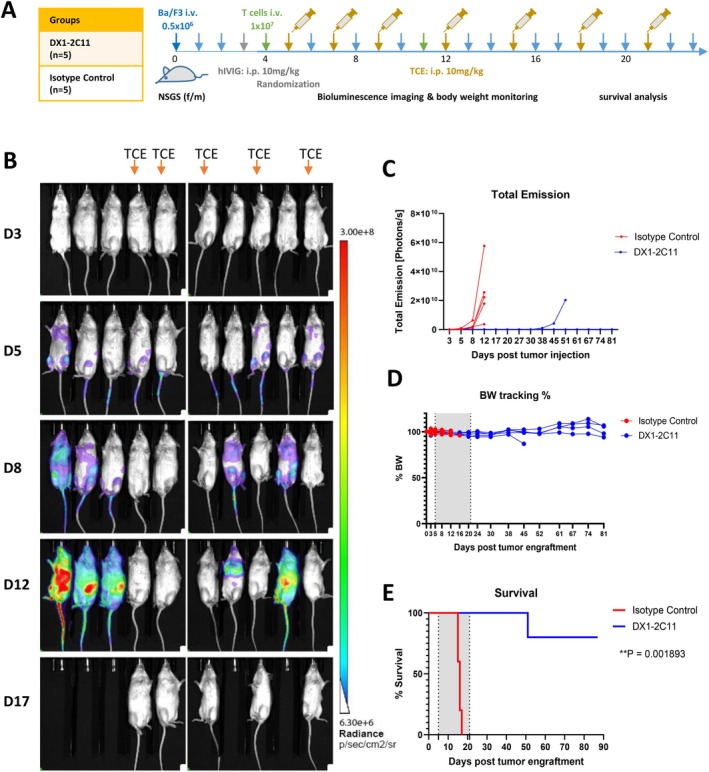
Long‐term in vivo efficacy of DX1‐2C11 in disseminated NSG mouse model engrafted with luciferase expressing Ba/F3‐MPL/CALR^del52^ cells. (A) Experimental timeline of the DX1‐2C11 efficacy study in NSG mice. 8–12‐week‐old NSG mice (5 mice per group) were i.v. injected with 0.5 million Ba/F3‐MPL/mutCALR Luc + cells and treated with 10 mg/mL TCE (DX1‐2C11 or isotype control) according to the illustrated timeline. (B) Visualization of tumor growth inhibition over time in treated mice after luciferin injection by LagoX and Aura (Spectral Instruments Imaging). (C) Quantification of total emission flux over time for animals treated with DX1‐2C11 or isotype control. (D) Body weight dynamics were not impacted by TCE treatment. (E) Kaplan–Meier survival analysis shown that DX1‐2C11 significantly prolonged the survival of the treated NSG mice. Treatment period were shaded in gray. **p < 0.001 was calculated by log‐rank (Mantel‐Cox) test. [Color figure can be viewed at wileyonlinelibrary.com]

Given that DX1‐2C11 efficiently depleted CALR‐mutant HSPCs and normalized hematopoiesis in immunocompetent mice, we next sought to investigate whether it could confer durable efficacy and prolong survival. To enable long‐term evaluation without confounding ADA immune responses, DX1‐2C11 was tested in the immune‐deficient NSG model. NSG mice were engrafted with luciferase expressing Ba/F3‐MPL/mutCALR cells on Day 0 and randomized by the luminance on Day 5 as indicated in Figure 6A. In total, seven doses of 10 mg/kg DX1‐2C11 or isotype control were administered intraperitoneally on Days 5, 7, 9, 12, 15, 18, and 21. Bioluminance imaging of five mice treated with DX1‐2C11 showed full depletion of Ba/F3 cells 3 days after treatment (Day 8), and 4 of them remained in complete remission after drug discontinuation (Figure 6B). Body weight differences were tracked throughout the experiment and no significant impact was observed between the DX1‐2C11 and the isotype control treated group (Figure 6D). The Ba/F3‐MPL/mutCALR cells proliferated and metastasized aggressively in control mice, resulting in the death of all animals within 17 days, with a median survival of only 16 days (Figure 6E). However, DX1‐2C11 significantly prolonged the survival of mice to over 87 days, with only 1 mouse relapsing and dying on Day 51 (Figure 6E). The remaining four mice survived without a sign of increased tumor growth till the end of the experiment, thus not yet having reached the median survival endpoint (Figure 6C,E). These data demonstrated effective disease control and significant survival benefits brought by the anti‐mutCALR/CD3ε bispecific T cell engager DX1‐2C11.

## 4 Discussion

Curative therapies for MPN require elimination of mutant hematopoietic stem cells. To date, only two TCE candidates have entered phase 1 clinical trials, and neither demonstrated specific mutant HSC depletion in preclinical studies [39, 40]. Here, we describe the development and preclinical evaluation of DX‐2C11, a murine TCE designed to redirect autologous mouse T cells for specific elimination of CALR mutated MPN stem cells. Our findings establish mutCALR as a viable target for TCE‐based therapies and provide a representative murine TCE platform along with a suite of in vivo models for thorough preclinical evaluation.

Although the membrane localization of mutCALR presents an attractive target for TCE therapy, it is not a transmembrane protein per se, but instead presented in complex with MPL via glycan‐ and protein‐mediated interactions [15, 16, 17, 18, 20, 21, 48, 49, 50, 51]. Accordingly, only CALR mutated, MPL positive cells are targets for DX1‐2C11. Despite potential concerns regarding epitope accessibility and MPL/mutCALR trafficking, our results demonstrated that T cell redirection via anti‐mutCALR TCEs constitutes an effective and selective therapeutic strategy.

We also discovered that soluble mutCALR attenuated DX1‐2C11 activity only at supraphysiologic concentrations (> 10 nM), suggesting that physiological levels [15] are unlikely to impair therapeutic efficacy. While only a monovalent 1 + 1 format was used in this study, future evaluation of bivalent or 2 + 1 formats may further enhance immune synapse formation and mitigate potential drug‐sink effects. Our in vivo data in transgenic mice further suggest that TCE efficacy is not compromised by the presence of soluble mutant CALR at concentrations comparable to those observed in patients [32, 50].

In immunocompetent transgenic models, DX1‐2C11 activated autologous T cells and induced rapid normalization of platelet counts, with confirmed depletion of mutant HSCs and myeloid progenitors in both primary and secondary recipients. For malignancies of the HSCs, these humanized transgenic models represent a uniquely informative platform since HSCs are defined biologically and only in vivo HSC assays allow conclusive preclinical investigation of cancer stem cell depletion. Long‐term efficacy was further demonstrated in a disseminated NSG model engrafted with luciferase‐labeled Ba/F3‐MPL/mutCALR cells, where multi‐dose DX1‐2C11 treatment resulted in marked tumor clearance and significantly prolonged survival compared with controls. Evaluation of durability in immunocompetent hosts was limited by anti‐drug antibody formation. Ongoing efforts therefore focus on murinizing the anti‐CD3 arm and developing humanized T‐cell models to enable extended in vivo testing of fully murine TCE surrogates corresponding to clinical drug candidates.

Although the therapeutic landscape of MPN is evolving rapidly, driven by expanded use of agents such as ropeginterferon alfa‐2b [52] and continued development of next‐generation JAK inhibitors [53] as well as novel agents targeting BET and BCL2 pathways [27, 28], true disease‐modification will likely require immunotherapies capable of eradicating mutant HSCs. In this context, our study provides the first preclinical proof‐of‐concept demonstrating specific and durable depletion of CALR‐mutant HSCs using a bispecific TCE, supporting further development of TCE‐based strategies for MPN.

## Author Contributions

S.X. and R.K. contributed to all assay design, data acquisition, interpretation, and analysis. T.W., C.V., and C.S. contributed to the data acquisition and interpretation of the in vivo studies. S.A. contributed to the epitope mapping ELISA. S.X. and R.K. wrote and revised the manuscript. T.W., C.V., C.S., and S.A. reviewed the manuscript.

## Funding

This work was supported by the Austrian Science Fund (P34451‐B) and MPN Research Foundation (2024 Challenge).

## Ethics Statement

All mouse husbandry and experimental activities were approved by the Medical University of Vienna in accordance with institutional guidelines for Good Scientific Practice, and authorized by the Austrian Federal Ministry of Education, Science and Research (FWF793A2003) under the animal license GZ2020‐0.448.862 and GZ2020‐0.406.011.

## Conflicts of Interest

The authors declare no conflicts of interest.

## Supporting information


**Appendix S1:** ajh70206‐sup‐0001‐supinfo.docx.

## Data Availability

The data that support the findings of this study are available from the corresponding author upon reasonable request.
