# Supplementary material for: A Murine Bispecific Antibody Efficiently Redirects T Cells Against Calr Mutated Stem Cells In Vivo
Source: Am J Hematol. 2026 Jan 14;101(4):697–709. doi: 10.1002/ajh.70206 (PMC12994124; doi:10.1002/ajh.70206)
Supplement: Supplementary file 1 — Appendix S1: ajh70206‐sup‐0001‐supinfo.docx. [file AJH-101-697-s001.docx]

**Supplemental Figures and Methods**

**A murine bispecific antibody efficiently redirects T cells against *Calr* mutated stem cells in vivo**

Shengen Xiong, Tamara Wais, Cecilia Varga, Christina Schueller, Sarada Achyutuni, Robert Kralovics*

*Department of Laboratory Medicine, Medical University of Vienna, Vienna, Austria*

***Correspondence:** Robert Kralovics, PhD**,** Department of Laboratory Medicine, Medical University of Vienna, Währinger Gürtel 18-20, 1090 Vienna, Austria; Email: [robert.kralovics@meduniwien.ac.at](mailto:Robert.kralovics@meduniwien.ac.at)

**Supplemental Table 1.** Engineered amino acid mutations in the murine TCE platform (Kabat numbering)

| **Name of Chains** | **Fc silencing mutations** | **Fc pairing mutations** |
| --- | --- | --- |
| Fab-Fc Heavy chain (Knob) | L234A, L235A, P329G | P354C, T366W |
| ScFv-Fc Heavy Chain (Hole) | L234A, L235A, P329G | Y349C, T366S, M368A, Y407V |
| Fab Light chain | Not Applicable | Not Applicable |

**Supplemental Table 2.** Binding kinetics of DX1-2C11 to mutCALR proteins.

| **Analyte** | **Antibody** | **K_a_ (M^-1^S^-1^)** | **K_d_ (S^-1^)** | **K_D_ (nM)** |
| --- | --- | --- | --- | --- |
| CALR-del52 | DX1-2C11 | 3.436 x 10^5^ | 7.266 x 10^-4^ | 2.115 ± 0.011 |
| CALR-ins5 | DX1-2C11 | 3.670 x 10^5^ | 4.239 x 10^-4^ | 1.155 ± 0.011 |

K_a_, association rate constant; K_d_, dissociation rate constant; K_D_, dissociation constant.


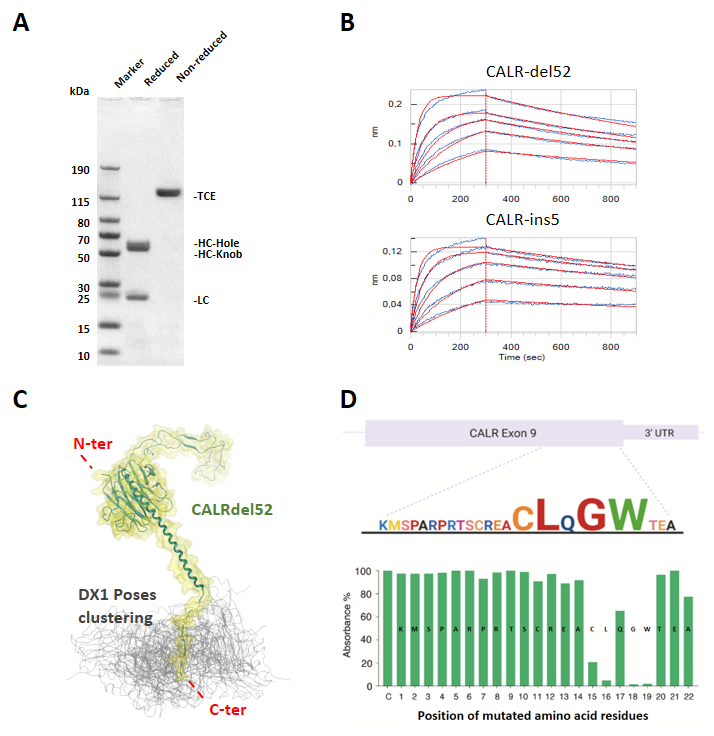


**Supplemental Figure 1. Epitope mapping of DX1-2C11 by *in silico* mapping and alanine scanning assay.** **A.** DX1-2C11 purity confirmed by Native PAGE electrophoresis after size exclusion chromatography (SEC) and ion exchange chromatography (IEX). **B.** The kinetic binding sensorgram of DX1-2C11 to CALR-del52 and CALR-ins5 using Octet Red96e. **C.** Clustering of the top poses via *in silico* molecular docking revealed the putative epitope of DX1-2C11 to be the last eight amino acids (CLQGWTEA) located in the C-terminus of mutant CALR. **D.** Alanine scanning ELISA assay confirmed that the epitope of DX1 is ‘CLQGW’. A peptide library of the last 22 amino acids each with a single alanine substitution (or glycine if it is alanine originally in that position) was used to determine the contribution for antibody-antigen binding.


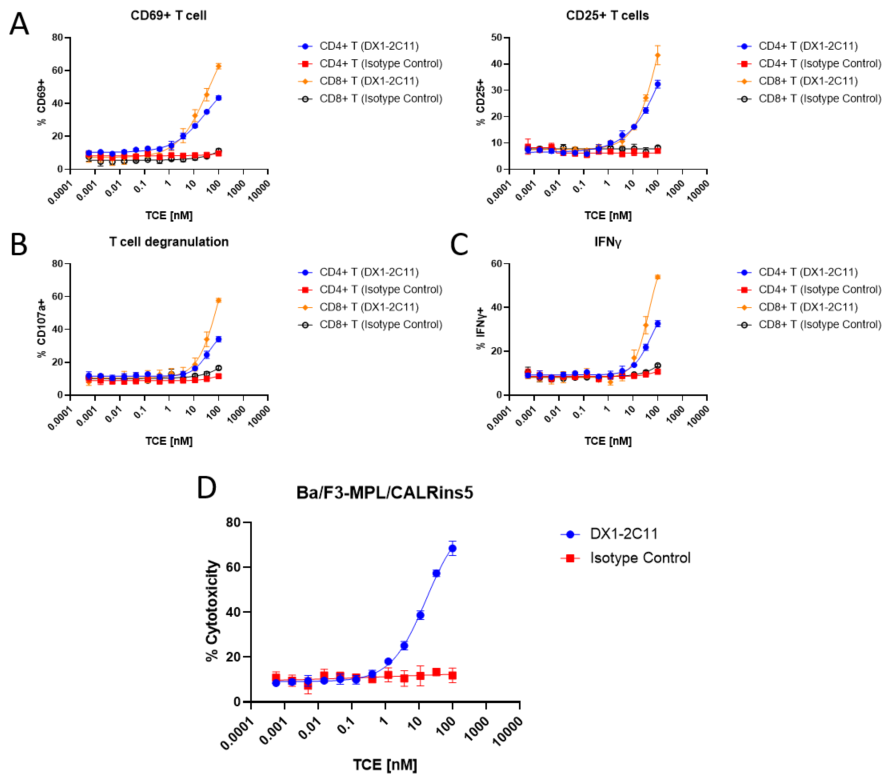


**Supplemental Figure 2. DX1-2C11 redirected mouse T cells against BaF3-MPL/CALRins5 cells. A.** T cell activation. **B.** T cell degranulation. **C.** IFNγ production. **D.** Ba/F3-MPL/CALRins5 cell killing after treatment with DX1-2C11 or TCE control molecules. Data represent the mean ± SD.


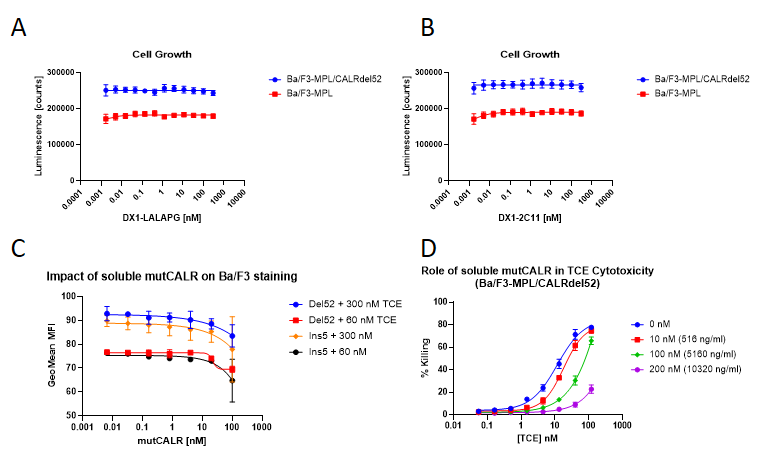


**Supplemental Figure 3. The anti-mutCALRxCD3e T cell engager DX1-2C11 does not inhibit the growth of Ba/F3 cells but the presence of excessive soluble CALRdel52 and CALRins5 influences the cytotoxicity of DX1-2C11.** **A-B.** CellTiter Glo assays showing no effects from DX1-LALAPG (**A**) or DX1-2C11 (**B**) on the cell growth of the Ba/F3-MPL and Ba/F3-MPL/mutCALR cells. Ba/F3-MPL cells or Ba/F3-MPL/CALRdel52 cells were seeded at the density of 40000 cells/well in 96 well plates. Serial diluted DX1-2C11 or DX1-LALAPG were added to the wells and incubated for 72h. Triplicates per plate and 3 plates for each drug. **C.** Impact of soluble mutCALR to the binding of DX1-2C11 on Ba/F3 cells at semi-saturated (60nM) and saturated (300nM) concentrations of TCE. **D.** Excessive soluble mutCALR attenuates the DX1-2C11 mediated T cell cytotoxicity against Ba/F3-MPL/CALRdel52 cells. Data represent the mean ± SD.


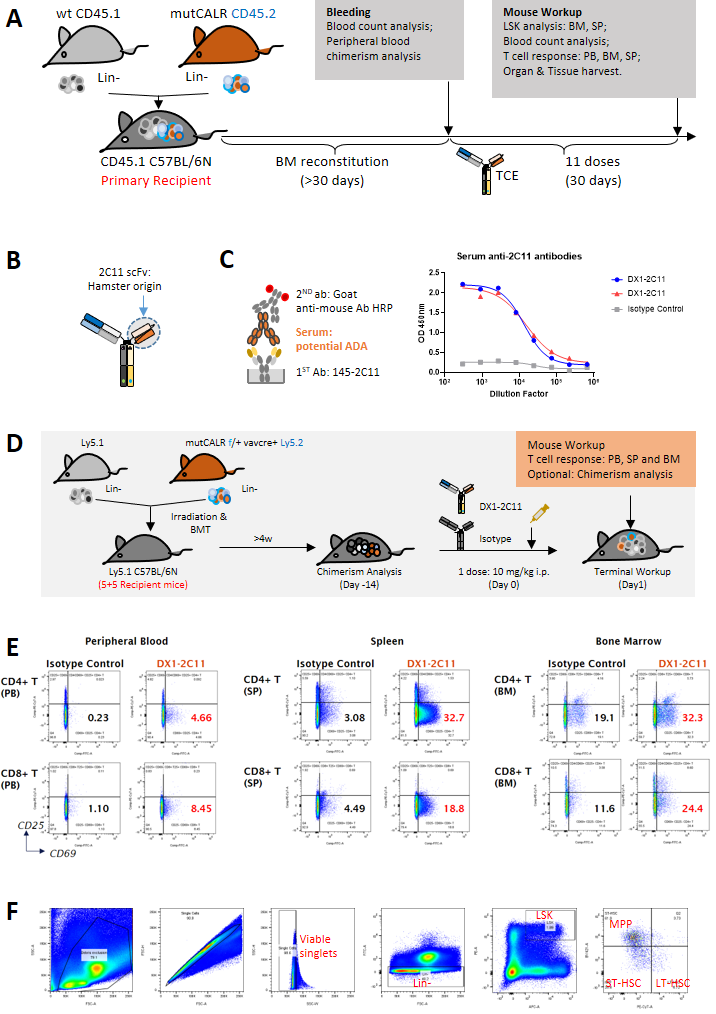


**Supplemental Figure 4.** **The long-term efficacy of DX1-2C11 was diminished by the 2C11 induced anti-drug antibody (ADA) effects while the short-term efficacy has been proven by T cell activation and mutant HSC depletion.** **A.** Schematic study design to investigate the long-term (30-day) efficacy of DX1-2C11 in VavCre CALR^del52/+^ mice. **B.** DX1-2C11 contains the anti-mouse CD3e single chain variable fragment (scFv) arm 2C11 from the hamster clone 145-2C11. **C.** The presence of high titer anti-[2C11] antibodies was detected in the sera of the mice treated with DX1-2C11 for 30 days via a specific ELISA assay using 145-2C11 monoclonal antibodies as the capture antibody. **D.** Schematic study design to quantify T cell activation 24h after TCE treatment in VavCre CALR^del52/+^ mice. Lin- bone marrow cells were isolated from wt CD45.1+ and VavCre CALR^del52/+^ CD45.2+ C57BL/6N mice. The bone marrow mixture were transplanted into lethally irradiated CD45.1+ C57BL/6N mice. Mice were randomized by peripheral CD45.2/CD45.1 chimerism analysis at 14 days ahead of TCE treatment. The mice were sacrificed 24h after TCE injection. Single cell suspensions from peripheral blood, spleenocytes and bone marrow cells were prepared for the analysis of T cell activation via flow cytometry. **E.** T cell activation in the peripheral blood, spleen and bone marrow. **F.** Gating strategies in the study to evaluate the efficacy of DX1-2C11 on day 7 in VavCre CALR^del52/+^ mice.


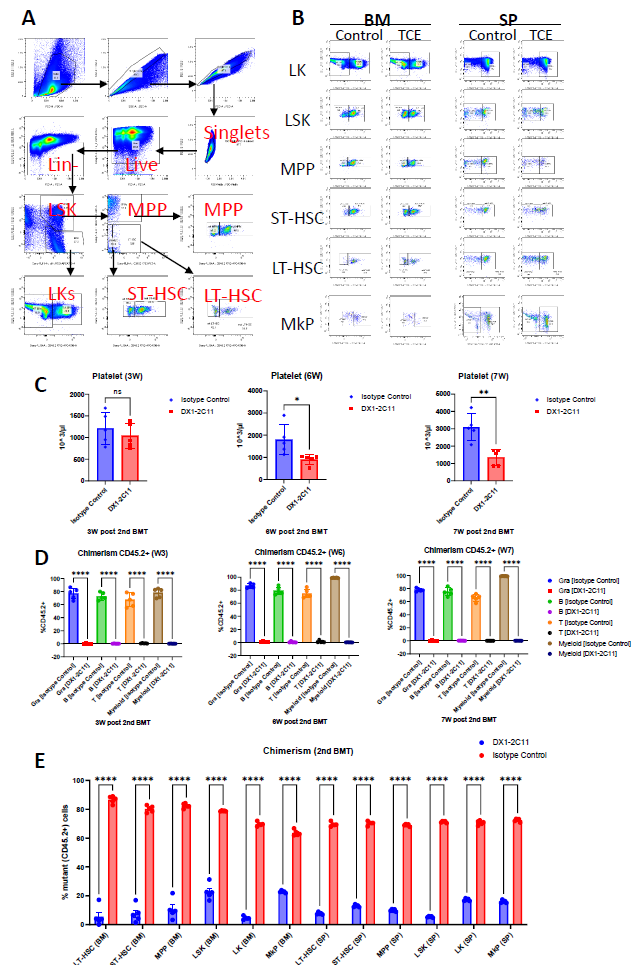


**Supplemental Figure 5. DX1-2C11 specifically depletes mutant (CD45.2^+^) HSPCs in the competitive bone marrow transplantation (cBMT) mouse study revealed that. A.** Gating strategies of various hematopoietic stem and progenitor cell compartments including LKs, LSKs, MPPs, ST-HSCs, LT-HSCs, and MkPs. **B.** Exemplified chimerism analysis (mutCALR [CD45.2^+^] / wt [CD45.1^+^]) in various HSPC compartments from the bone marrow (BM) and spleen (SP). **C.** Comparison of platelet counts in peripheral blood in the recipient mice with secondary bone marrow transplantation. **D.** CD45.2/CD45.1 chimerism in the B cells, T cell, granulocyte, and pan myeloid cell compartments in the peripheral blood of the secondary recipient mice at week 3, 6 and 7. **E.** CD45.2/CD45.1 chimerism analysis of hematopoietic stem cells and progenitors in bone marrow (BM) and spleen (SP) in the secondary recipient mice. Significance was determined using a 2-way ANOVA analysis. ∗*P* < 0.05, ∗∗*P* < 0.01, ∗∗∗*P* < 0.001.

**Supplemental Methods**

**Protein Production and Purification**

Gene cassettes of the three TCE chains were cloned into the pcDNA3.1 vector and then transiently transfected into Expi293F cells (A14635, Gibco) for protein production. Cells were transfected at a density of 3 million per ml with engineered chains vectors (pcDNA3.1 plasmids) as instructed by the manual of the kit. Then TCEs were purified from the clarified supernatant by protein A chromatography (HiTrap MabSelect PrismA column, 17549851, Cytiva). Protein aggregates and TCE related impurities were removed via size exclusion chromatography (SEC) using HILOAD® 16/600 SUPERDEX® 200 PG column (28989335, Cytiva) and cation exchange chromatography (CEX) using Mono S 4.6/100 PE column (17518001, Cytiva) to reach high monodispersity. Recombinant CALRdel52 or CALRins5 proteins were engineered with a c-terminal Twin-Strep tag®. Proteins were produced by transient transfection of pcDNA3.1 vectors into Expi293F cells and subsequently purified by affinity chromatography using Strep-Tactin® 4Flow® high-capacity FPLC columns (IBA Life sciences, 2-1258-001) in combination with the Strep-Tactin®XT 4Flow® Starter Kit (IBA Life sciences, 2-5998-000).

Protein purity was assessed using SDS-PAGE (NuPAGE™ Bis-Tris Mini Protein Gels, 4–12%, 1.0–1.5 mm; Invitrogen, NP0321BOX) followed by Coomassie Brilliant Blue staining.

***In silico* modelling, docking and epitope analysis**

The structure of mutant CALR proteins (CALRdel52 and CALRins5) were modelled using AlphaFold 2. The structure of MPL/CALRdel52 complex was previously simulated and reported. The structure of the DX1 antibody and the DX1-2C11 TCE was modelled through a multi-step hybrid process of homology, database comparison and energy minimization using Rosetta Antibody (Framework: 3KS0.J and 3KS0.K; CDR L1: 3ks0.J, L2: 6N5D.N, L3: 6N5D.N, H1: 1KCR.H, H2: 7U09.H, H3: 7JTR.H D1). Docking simulations were performed to the CALRdel52 monomer as well as the MPL/CALRdel52 tetramer structures using Rosetta Dock. The epitope of DX1 was first predicted based on binding interactions observed in the top-ranked docking poses. This prediction was subsequently validated by an epitope mapping assay employing the alanine walking peptide library described as following.

**Epitope mapping via alanine walking peptide library**

Firstly, a decapeptide library covering the neoantigen region of the mutant CALR proteins were used to screen for the mutCALR epitope of DX1. The binding epitope of DX1 was narrowed down to the last 10 amino acids (EACLQGWTEA) of the shared mutant region on mutant calreticulin.

An alanine-walking library of the last 22 amino acids (KMSPARPRTSCREACLQGWTEA) was used to confirm critical amino acids contributing to DX1 binding. Each 22-mer peptide contains a single amino acids replaced by alanine or glycine if it is alanine originally (see Table 1).

**Table 1: The alanine walking peptide library of the 22 c-terminal amino acids shared by all mutant calreticulin proteins.**

| Control | KMSPARPRTSCREACLQGWTEA |
| --- | --- |
| Analogue 1 | AMSPARPRTSCREACLQGWTEA |
| Analogue 2 | KASPARPRTSCREACLQGWTEA |
| Analogue 3 | KMAPARPRTSCREACLQGWTEA |
| Analogue 4 | KMSAARPRTSCREACLQGWTEA |
| Analogue 5 | KMSPGRPRTSCREACLQGWTEA |
| Analogue 6 | KMSPAAPRTSCREACLQGWTEA |
| Analogue 7 | KMSPARARTSCREACLQGWTEA |
| Analogue 8 | KMSPARPATSCREACLQGWTEA |
| Analogue 9 | KMSPARPRASCREACLQGWTEA |
| Analogue 10 | KMSPARPRTACREACLQGWTEA |
| Analogue 11 | KMSPARPRTSAREACLQGWTEA |
| Analogue 12 | KMSPARPRTSCAEACLQGWTEA |
| Analogue 13 | KMSPARPRTSCRAACLQGWTEA |
| Analogue 14 | KMSPARPRTSCREGCLQGWTEA |
| Analogue 15 | KMSPARPRTSCREAALQGWTEA |
| Analogue 16 | KMSPARPRTSCREACAQGWTEA |
| Analogue 17 | KMSPARPRTSCREACLAGWTEA |
| Analogue 18 | KMSPARPRTSCREACLQAWTEA |
| Analogue 19 | KMSPARPRTSCREACLQGATEA |
| Analogue 20 | KMSPARPRTSCREACLQGWAEA |
| Analogue 21 | KMSPARPRTSCREACLQGWTAA |
| Analogue 22 | KMSPARPRTSCREACLQGWTEG |

**Cell lines and cell culture**

Ba/F3-MPL or Ba/F3-MPL/mutCALR cell lines were generated in house via CRISPR engineering from the parental Ba/F3-MPL cell line. The Ba/F3 cells were cultured in RPMI 1640 with 10% fetal bovine serum in addition with 2% WEHI-3 supernatant, 100 U/ml penicillin and 100 ug/ml streptomycin at 37°C in a 5% CO_2_ incubator.

**Bio-layer interferometry (BLI) to determine affinities to mutCALR and CD3ε (Octet)**

Binding affinities and kinetics of the TCEs to CALRdel52, CALRins5 and mouse CD3ε were measured using the Octet Red96e system (Fortebio, Sartorious). Recombinant CALRdel52 or CALRins5 proteins were biotinylated and dialyzed using the EZ-Link™ Sulfo-NHS-SS-Biotinylation Kit (21445, Thermo Scientific) according to the manufacturer’s manual. Number of biotins per molecule were quantified via the HABA assay and the products were undergone DPBS buffer exchange to remove residual reagents. Biotinylated recombinant mouse CD3e proteins were ordered from commercial supplier (CDG-M58D2-25ug, AcroBiosystems).

The monovalent 1+1 format of our TCE platform allows direct measurement of kinetic constants without firstly immobilize the TCEs on the biosensors. The Streptavidin (SA) biosensors (Cat#18-5020, ForteBio) were rehydrated for 10min at room temperature. Baseline measurement lasts for 60s followed with a loading step of dipping the SA sensors into 10 ng/ml biotinylated CALRdel52, CALRins5 and mouse CD3εδ (10ug/ml, 200ul/well) for 120s. Continued with an additional of baseline reading in assay buffer for 60s, the binding dynamics of TCEs (100 nM, 50 nM, 25 nM, 12.5 nM and blank) were measured for 300s. Lastly, the biosensors undergone the dissociation step (300s) in blank assay buffer. Data analysis was performed using the Octet® Data Analysis software (version 8.1, ForteBio).

**Cell proliferation assay**

The impact of TCEs or monoclonal antibodies on the growth of Ba/F3-MPL and Ba/F3-MPL/CALRdel52 cells was assessed using a CellTiter-Glo® Luminescent Cell Viability Assay (Promega). The Ba/F3 cells were seeded at a density of 40000 cells per 100ul Ba/F3 cell culture medium per well into 96 well micro-titer plates. TCEs or DX1 monoclonal antibodies were serial diluted with the same Ba/F3 cell culture medium. The serial diluted antibodies were then added 100ul per well into the wells seeded with Ba/F3 cells. The plates were cultured in an incubator at 37°C, 5% CO_2_ for 72h. The cells were gently mixed and then 50ul/well mixture were transferred to a white 96-well micro-titer plate. 50ul/well CellTiter-Glo reagent well added to the white plates and these plates were mildly shaked, incubated in the dark at room temperature for 10min before luminescence quantification using Synergy H1 (BioTek).

**T cell and Ba/F3 cell staining**

Mouse T cells, Ba/F3-MPL or Ba/F3-MPL/mutCALR cells were seeded at the cell density of 2x10^5^ cells per well in round-bottom 96 well plates. The plates were centrifuged at 500 g for 5 min to remove supernatant and the cell pellets were resuspended with 100ul/well FACS buffer (DPBS containing 1% BSA) containing 1 ug/200-ul TruStain FcX™ (anti-mouse CD16/32) antibody. After incubation for 10 minutes at room temperature, the cells were centrifuged and then stained with DX1-2C11 or isotype control at 3-fold serial diluted concentrations starting from 100 nM for 45min at 4 °C. The cells were washed with FACS buffer twice and resuspended in 100ul FACS buffer containing Zombie Aqua Fixable Viability dye (1:1000 diluted with FACS buffer) and the anti-G4S-AF647 antibody (Cell Signaling, 69782S) (1:100 diluted in FACS buffer) for 30min at 4 °C in the dark. Samples in this study were acquired using flow cytometers including MACS quant Analyzer 16 (Miltenyi Biotec), CytoFlex LX (Beckman Coulter) and BD LSRFortessa. Viable single cells were gated and the GeoMean fluorescent intensity (GeoMFI) values were calculated using FlowJo and the curves were plotted by GraphPad Prism.

**Table 2: Reagents used in the T cell and Ba/F3 cell staining assays.**

| **Name** | **Clone** | **Source** | **Catalog No.** | **Dilution** |
| --- | --- | --- | --- | --- |
| TruStain FcX™ (anti-mouse CD16/32) Antibody | N/A | Biolegend | 101320 | 1ug/100ul |
| G4S Linker (E7O2V) Rabbit mAb (Alexa Fluor® 647 Conjugate) | E7O2V | Cell Signaling | 69782S | 1:1000 |
| Zombie Aqua™ Fixable Viability Kit | N/A | Biolegend | 423102 | 1:1000 |

**TCE binding competition assay (with soluble mutCALR)**

Ba/F3 cells were blocked with 100ul/well FACS buffer (DPBS supplemented with 1% BSA) containing 1 ug/200-ul TruStain FcX™ (anti-mouse CD16/32) antibody. Cells were seeded into 96-well V bottom plates and incubated with 100ul/well DPBS containing soluble mutCALR proteins (CALRdel52 and CALRins5) at concentrations of 100 nM, 33.3 nM, 11.1 nM, 3.7 nM, 1.2 nM, 0.4 nM and 0 nM, in the presence of 300 nM or 60 nM TCEs. Following a 30 min incubation at 4 °C, cells were washed twice with DPBS containing 1% BSA. Samples were resuspended with 100ul/well of staining buffer containing the secondary goat anti-mouse IgG2a-Alexa Fluor 647 antibodies (1:4000 dilution) for 30 minutes at 4°C. The cells were washed twice with FACS buffer. Flow cytometry was performed to assess the TCE binding signals determined by using the GeoMFI values from single-parameter histograms.

**T cell redirection assay**

Pan-mouse T cells isolated from mouse spleenocytes were expanded for 3-5 days and rested for another 2 days using the commercial kit (Dynabeads™ Mouse T-Activator CD3/CD28 for T-Cell Expansion and Activation kit, Gibco). The following flow cytometry panel (see Table 3) was used to quantify T cell activation in the assays using Ba/F3 cells and bone marrow cells as targets. Live/Dead staining was performed after initial staining with all the membrane markers. Fixation and cell permeabilization were conducted before the intracellular staining with anti-CD107a and anti-IFNγ antibodies. Data was analyzed using FlowJo.

**Table 3. Flow cytometry panel used for the measurement of T cell activation in vitro**

| **Marker/channel** | **Clone** | **Supplier** | **Catalog No.** | **Dilution** |
| --- | --- | --- | --- | --- |
| TCRβ-APC/Fire™ 750 | H57-597 | Biolegend | 109246 | 1: 500 |
| CD4-eFluor450 | GK1.5 | eBiosciences | 48-0041-82 | 1: 1000 |
| CD8a-BV605 | 53-6.7 | Biolegend | 100743 | 1: 1000 |
| CD69-FITC | H1.2F3 | Biolegend | 104505 | 1: 1000 |
| CD25-PE-Cy7 | PC61.5 | EnquireBio | QAB34-PE7-100UG | 1: 1000 |
| IFN-gamma-PE | XMG1.2 | Invitrogen | 12-7311-82 | 1: 1000 |
| CD107a- APC-eFluor660 | eBio1D4B | Invitrogen | 5-1071-80 | 1: 1000 |
| 7AAD | N/A | Biolegend | 420403 | 1: 1000 |
| Anti-Mouse CD16/32 | N/A | InVivoMab | BE0307 | 1: 300 |

**Competitive T cell redirection assay (with soluble mutCALR)**

Soluble mutCALR proteins (CALRdel52) were added the co-cultures of T cells, Ba/F3 cells to reach the final concentrations of 10 nM, 100 nM, and 200 nM. The same flow cytometry panel listed on Table 3 has been used to quantify T cell activation and tumor killing.

**Mouse workup and sample preparation for flow cytometry analysis**

Peripheral blood was withdrawn into K3EDTA (Ref. 450531, Greiner) or CAT Serum Separation (Ref. 450533, Greiner) MiniCollect tubes via tail vein bleeding. Mice were euthanized and dissected for the collection of livers, spleens, sternums, femurs and tibias. Bone marrow cells were isolated by aseptically crushing the bones. Splenocytes and bone marrow cells were prepared by mechanically dissociating and filtering the tissues through sterile 70-µm cell strainers to generate single-cell suspensions. Cells were pelleted by centrifugation at 500 x g for 5 min, resuspended in 1x RBC lysis buffer and incubated at room temperature for 5 min to remove red blood cells. Following lysis, cells were washed with DPBS, and excess samples were cryopreserved for downstream analyses.

**Table 4. Flow cytometry panels used for the in vivo studies using the VavCre CALR^del52/+^ or VavCre CALR^del52/del52^ mouse models**

| **Table 4A. Panel for Chimerism analysis in peripheral Blood** | | | | |
| --- | --- | --- | --- | --- |
| **Marker/channel** | **Clone** | **Supplier** | **Catalog No.** | **Dilution** |
| CD45.2-efluor450 | 104 | Invitrogen | 48-0454-82 | 1: 1000 |
| CD3e-PerCP/Cy5.5 | 145-2C11 | Biolegend | 100328 | 1: 1000 |
| CD11b-FITC | M1/70 | Biolegend | 101206 | 1: 1000 |
| CD19-PE/Cy7 | eBio1D3 | Invitrogen | 25-0193-82 | 1: 1000 |
| 7-AAD | N/A | Biolegend | 420403 | 1: 1000 |
| CD45.1-PE | A20 | Invitrogen | 12-0453-83 | 1: 1000 |
| GR1-APC/H7 | 1A8 | BD Pharmingen | 565369 | 1: 1000 |
| Ter119-APC | TER-119 | Biolegend | 116212 | 1: 1000 |
| Anti-Mouse CD16/32 | N/A | InVivoMab | BE0307 | 1: 300 |

| **Table 4B. T cell activation panel** | | | | |
| --- | --- | --- | --- | --- |
| **Marker/channel** | **Clone** | **Supplier** | **Catalog No.** | **Dilution** |
| TCRβ-APC/Fire™ 750 | H57-597 | Biolegend | 109246 | 1: 500 |
| CD4-eFluor450 | GK1.5 | eBiosciences | 48-0041-82 | 1: 1000 |
| CD8a-BV605 | 53-6.7 | Biolegend | 100743 | 1: 1000 |
| CD69-FITC | H1.2F3 | Biolegend | 104505 | 1: 1000 |
| CD25-PE-Cy7 | PC61.5 | EnquireBio | QAB34-PE7-100UG | 1: 1000 |
| Anti-Mouse CD16/32 | N/A | InVivoMab | BE0307 | 1: 300 |

| **Table 4C. HSPC depletion analysis panel** | | | | |
| --- | --- | --- | --- | --- |
| **Marker/channel** | **Clone** | **Supplier** | **Catalog No.** | **Dilution** |
| Anti-Mouse CD16/32 | N/A | InVivoMab | BE0307 | 1: 300 |
| Lineage-FITC | 17A2, RA3-6B2, M1/70, TER-119, RB6-8C5 | eBiosciences | 22-7770-72 | 1: 1000 |
| Sca-1-BV711 | D7 | BD Horizon | 563992 | 1: 1000 |
| c-Kit-APC/efluor780 | 2B8 | invitrogen | 47-1171-82 | 1: 1000 |
| CD48-APC | HM48-1 | eBiosciences | 17-0481-80 | 1: 1000 |
| CD150-PE/Cy7 | mShad150 | invitrogen | 25-1502-82 | 1: 1000 |
| CD41-PE | MWR REG30 | Biolegend | 133906 | 1: 1000 |
| CD34-BV421 | RAM34 | BD Horizon | 562608 | 1: 1000 |
| 7AAD | N/A | Biolegend | 420403 | 1: 1000 |

| **Table 4D. The definition of various subpopulation of mouse HSPCs in our study.** | |
| --- | --- |
| Lin- | Lin- |
| LK | Lin- cKit+ |
| LSK | Lin- Sca1+ cKit+ |
| SLAM | LSK CD48- CD150+ |
| LT-HSC | LSK CD48- CD150+ CD34- |
| ST-HSC | LSK CD48- CD150+ CD34+ |
| MkP | LK Sca-1- CD150+ CD41+ |

**Table 5. Flow cytometry panels used for the in vivo study using the competitive bone marrow transplantation (cBMT) mouse model**

| **Table 5. HSPC chimerism analysis panel** | | | | |
| --- | --- | --- | --- | --- |
| **Marker/channel** | **Clone** | **Supplier** | **Catalog No.** | **Dilution** |
| Anti-Mouse CD16/32 | N/A | InVivoMab | BE0307 | 1: 300 |
| Lineage-FITC | 17A2, RA3-6B2, M1/70, TER-119, RB6-8C5 | eBiosciences | 22-7770-72 | 1: 1000 |
| Sca-1-BV711 | D7 | BD Horizon | 563992 | 1: 1000 |
| c-Kit-APC/efluor780 | 2B8 | invitrogen | 47-1171-82 | 1: 1000 |
| CD48-APC | HM48-1 | eBiosciences | 17-0481-80 | 1: 1000 |
| CD150-PE/Cy7 | mShad150 | invitrogen | 25-1502-82 | 1: 1000 |
| CD41-PE | MWR REG30 | Biolegend | 133906 | 1: 1000 |
| CD34-BV421 | RAM34 | BD Horizon | 562608 | 1: 1000 |
| CD135-PerCP/efluor710 | A2F10 | Invitrogen | 46-1351-82 | 1: 1000 |
| CD45.1-BV605 | A20 | Biolegend | 110737 | 1: 1000 |
| CD45.2-AlexaFluor700 | 104 | eBiosciences | 56-0454-82 | 1: 1000 |
| 7AAD | N/A | Biolegend | 420403 | 1: 1000 |
